# Combination of expression levels of miR-21 and miR-126 is associated with cancer-specific survival in clear-cell renal cell carcinoma

**DOI:** 10.1186/1471-2407-14-25

**Published:** 2014-01-15

**Authors:** Daniel Vergho, Susanne Kneitz, Andreas Rosenwald, Charlotte Scherer, Martin Spahn, Maximilian Burger, Hubertus Riedmiller, Burkhard Kneitz

**Affiliations:** 1 Department of Urology and Paediatric Urology, Julius-Maximilians-University Medical Centre of Würzburg, Würzburg, Germany; 2 Physiological Chemistry I, Biocentre, University of Würzburg, Würzburg, Germany; 3 Department of Pathology, University of Würzburg, Würzburg, Germany; 4 Department of Urology, University of Bern, Bern, Switzerland

**Keywords:** Renal cell carcinoma, RCC, Kidney cancer, miRNA, miR-21, miR-126, Prognosis, Profiling, Biomarker, Tumour markers

## Abstract

**Background:**

Renal cell carcinoma (RCC) is marked by high mortality rate. To date, no robust risk stratification by clinical or molecular prognosticators of cancer-specific survival (CSS) has been established for early stages. Transcriptional profiling of small non-coding RNA gene products (miRNAs) seems promising for prognostic stratification. The expression of miR-21 and miR-126 was analysed in a large cohort of RCC patients; a combined risk score (CRS)-model was constructed based on expression levels of both miRNAs.

**Methods:**

Expression of miR-21 and miR-126 was evaluated by qRT-PCR in tumour and adjacent non-neoplastic tissue in n = 139 clear cell RCC patients. Relation of miR-21 and miR-126 expression with various clinical parameters was assessed. Parameters were analysed by uni- and multivariate COX regression. A factor derived from the z-score resulting from the COX model was determined for both miRs separately and a combined risk score (CRS) was calculated multiplying the relative expression of miR-21 and miR-126 by this factor. The best fitting COX model was selected by relative goodness-of-fit with the Akaike information criterion (AIC).

**Results:**

RCC with and without miR-21 up- and miR-126 downregulation differed significantly in synchronous metastatic status and CSS. Upregulation of miR-21 and downregulation of miR-126 were independently prognostic. A combined risk score (CRS) based on the expression of both miRs showed high sensitivity and specificity in predicting CSS and prediction was independent from any other clinico-pathological parameter. Association of CRS with CSS was successfully validated in a testing cohort containing patients with high and low risk for progressive disease.

**Conclusions:**

A combined expression level of miR-21 and miR-126 accurately predicted CSS in two independent RCC cohorts and seems feasible for clinical application in assessing prognosis.

## Background

The incidence of RCC is increasing annually by about 2% [1]. Over 200 000 new cases are diagnosed per year and more than 100 000 related deaths occur globally [2]. RCC is marked by adverse tumour biology and its CSS ranks lowest among urological malignancies [3]. RCC is clinically demanding due to its prognostic heterogeneity. The establishment of concepts of adjuvant therapy has been hindered by lacking reliability of prediction of outcome by both clinical and molecular parameters. Therefore, identification of novel markers is warranted for tailoring therapy and follow-up. One current approach for molecular tumour characterization is profiling of microRNA (miR) expression [4]. MiRs are small noncoding RNA strands posttranscriptionally regulating gene expression and appearing to be modulators of urologic cancers [5]. Among the large number of miRs, miR-21 and miR-126 have received special attention because of their relationship with multiple cancer entities. Upregulation of miR-21 has been reported e.g. in breast, gastric and lung cancer [4]. A respective role has also been suggested for urological malignancies. In prostate cancer, elevated expression of miR-21 alone was shown to convey castration resistance [6]. In RCC, several studies describe upregulation of miR-21 [7,8] and recently, association with reduced survival [9], indicating a pathogenetical role of miR-21 as a so-called oncomiR.

Such role has also been suggested for miRNA-126, which is mapped within its host gene EGFL-7 (epidermal growth factor like-7) and is highly expressed in vascular endothelial cells [10]. By regulating the VEGF (vascular endothelial growth factor) pathway miR-126 plays an important role in angiogenesis, lymphangiogenesis and vessel integrity in endothelial cells as well as in cancer cells [11]. In several studies miR-126 was reported to act as a tumour suppressor and was shown to be downregulated in various cancer types including breast, gastric, prostate cancer and RCC [11,12]. In non-small cell lung and oral squamous cell cancer downregulation of miR-126 was related to poor survival suggesting miR-126 to be prognostic [13,14]. In metastatic colorectal cancer miR-126 was related to the response of treatment with capecitabine and oxaliplatin [15]. In RCC miR-126 is described to play a role in molecular classification of different subtypes [12] and recently, association of downregulation with progression was supposed [16,17].

While dysregulation of miR-21 and miR-126 has been linked to metastasis and progression in many cancer types, to date data on RCC are scarce. To assess a potential role of these miRs as prognostic molecular markers in RCC we analysed the expression of both miRs in 139 clear cell RCC specimens aiming at clinical application as molecular markers.

## Methods

### Patients and tissue sample preparation

Fresh frozen samples of clear-cell RCC and adjacent histologically benign renal tissue of patients undergoing radical nephrectomy or nephron-sparing surgery at the Department of Urology and Paediatric Urology of the Julius- Maximilians-University Medical Centre Würzburg between 2006–2010 were included in the study. Fresh specimens were collected, snap frozen in liquid nitrogen immediately after resection, and directly stored at -80C until RNA extraction was performed. Samples from cancerous areas were isolated from non-necrotic parts of the tumour tissue. Tumour classification and staging were performed according to the 2004 World Health classification and the 2002 TNM System. The study was approved by the Ethical Review Board of the Julius-Maximilian-University Würzburg (no. 136/08) and written informed consent was obtained from all patients.

### RNA extraction and quantitative real time PCR

Total RNA from frozen tissue was isolated using the miRNAeasy kit (Quiagen, Hilden, Germany) according to the manufacturer’s instructions. RNA concentration and A260/280 ratio were analysed with a Nano Drop ND-100 spectrometer (NanoDrop Technologies, Wilmington) and RIN (RNA Integrity Numbers) and calculated with a Bioanalyzer. RNA samples showing RIN < 6.0 were excluded from further analysis. The resulting miRNA was retained for quantitative Real Time PCR (qRT-PCR).

QRT-PCR was performed using TaqMan Micro Array assays (Applied Biosystems) as described previously. 5 ng total RNA was used for microRNA-specific reverse transcription as recommended by the manufacturer for miR-21 and miR-126. Cycling conditions were chosen according to manufacturer’s protocols. All reactions were performed in triplicates and samples showing SD > 0.5 were excluded. Relative expression values of miRs were normalized to small nuclear RNA (RNU6b) previously described as reference gene [18]. ΔC_t_ for tumour samples and adjacent non neoplastic tissue of the two miRs were performed by the comparative C_t_ method. Relative over- or underexpression of miRs in tumours compared to the normal adjacent kidney tissue was obtained by the ΔΔC_t_ method assuming equal RNA concentrations and complete efficiency of qRT-PCR as described previously [18]. All samples characterized by expression levels of RNU6B > 30 C_t_ were excluded from further analysis (Additional file 1: Table S1).

### Statistics, computational analysis and combined risk score calculation

Thresholds for dichotomising relative expressions of miR-21 and miR-126 were determined by receiver operating characteristic (ROC) curve, based on CSS. Impact of clinic-pathological parameters, miR-21 and miR-126 on CSS was assessed by uni- and multivariate COX regression analysis (R package, Thernaux, 2000). Calculation of a CRS of miR-21 and miR-126 was implemented as proposed by Lossos et al. [19]. Therefore, a factor derived from the z-score, resulting from the COX model, was determined for both miRs separately and the relative expression multiplied by this factor resulting in the formula (−2.1 × miR-126) + (2.6 × miR-21). The negative factor indicates that higher expression correlates with longer survival, whereas the positive factor correlates with shorter survival. A cut-off for the risk score was again determined by ROC curve. The best fitting COX model was selected by measuring the relative goodness-of-fit with the Akaike information criterion (AIC). Differences in mean between miR- expression and clinical parameters were analysed by Student’s t-test and ANOVA.

## Results

### Expression of miR-21 and miR-126 in RCC

**Figure 1:**
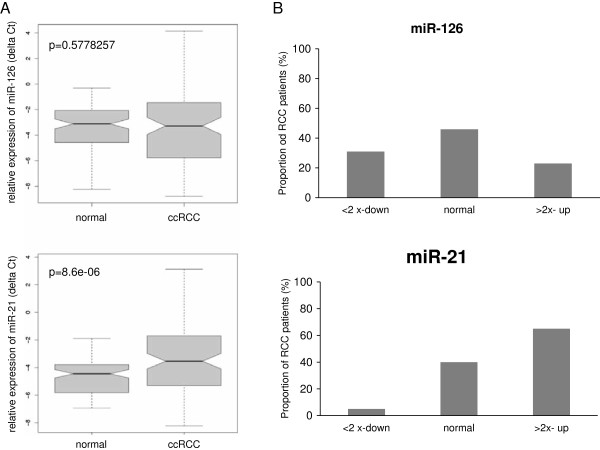
**Expression of miR-126 and miR-21 in RCC.** Box-Whisker-Plot : **A)** Relative expression of miR-21 and miR-126 in RCC and normal renal tissue (n = 103). MiR-21 expression is significantly higher in RCC (p < 0,001), but overall miR-126 expression is not changed (p = 0.57) in RCC compared to the expression of control tissue. Expression was analysed by qRT-PCR and normalized against RNU6b. P-values were calculated by unpaired students t-test. **B)** Proportion of RCC patients with dysregulation of miR-21 and miR-126: Expression of miRs in tumour and control tissue was verified by qRT-PCR in triplicates. MiR expression ratio of each tumour specimen compared to the expression in corresponding adjacent normal tissue was calculated by the ΔΔC_t_ method. All patients were divided into three groups by > < 2 fold up- or down-regulation of miR-21 or miR-126 in RCC.

As previously described by other studies miR-21 and miR-126 was expressed in normal kidney tissue and in RCC samples (Additional file 1: Table S1). Moreover, we found a significant upregulation of miR-21 in RCC (Figure 1A) compared to adjacent renal tissue by qRT-PCR. Clinical and pathological characteristics of the used collective are summarized in Table 1. The ΔΔCt method demonstrated miR-21 to be upregulated more than two-fold in 65% of the RCC cases. In addition to the known oncomiR miR-21 we analysed expression of miR-126 in our study collective. As shown in Figure 1A no significantly different overall expression of miR-126 between malign and benign samples was observed. However, the standard deviation (SD) of miR-126 expression in RCC samples showed greater variation compared to the SD in histologically benign tissue (Figure 1A). The comparative ΔΔCt method showed a more than twofold downregulation and a more than two-fold upregulation of miR-126 in 36 (35%) and 22 (22%) RCC samples, respectively (Figure 1B). From these results we concluded that miR-126 was silenced or upregulated in different subgroups of the RCC study cohort resulting in comparable mean expression between RCC cases and adjacent normal renal tissue.

**Table 1:** Clinical and pathological patient characteristics (n = 103)

**Clinical/ patholigical features**	**n**	
Age, years (range)	65 (32–91)	
Follow up, months (st. dev)	33 (± 15, 2)	
Pathological tumor stage		
pT1a	27, (26, 2%)	
pT1b	28 (27, 2%)	
pT2	11 (10, 7%)	
pT3a	7 (6, 8%)	
pT3b	28 (27, 2%)	
pT3c	2 (1, 9%)	
Grading		
1	12 (11, 7%)	
2	69 (66, 9%)	
3	22 (21, 4%)	
Metastasis at time of surgery		
No	88 (84, 5%)	
Yes	16 (15, 5%)	
Surgery		
nephron sparing	26 (25, 2%)	
	pT1a	67% (18 of 27 patients)
	pT1b	29% (8 of 28 patients)
	pT2-3c	0% (0 of 48 patients)
nephrectomy	77 (74, 8%)	
Clinical failure		
No	82 (79, 6%)	
Yes	21 (20, 4%)	
Cancer related death		
No	87 (84, 5%)	
Yes	16 (15, 5%)	

### Association of miR-21 and miR-126 expression with clinical parameters in RCC

**Figure 2:**
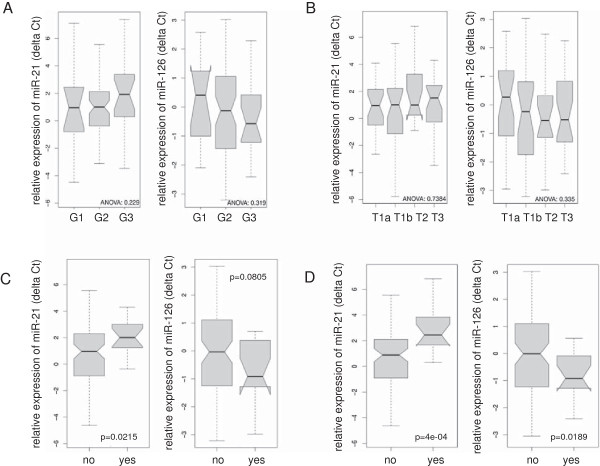
**MiR-21 and miR-126 expression in association to clinical parameters.** Box-Whisker-Plot: relative expression of miR-21 and miR −126 was analysed by the ΔΔC_t_ method in RCC cases and subsequently the cases were divided into subgroups based on tumor grade **(A)**, T stage **(B)** the presence of synchrone metastasis **(C)** or cancer related death (CRD) **(D)**. P-values were calculated by students paired t-test **(C and D)** or by ANOVA **(A and B)**.

To test a potential clinical relevance of miR-21 or miR-126, we analysed their expression in different risk groups stratified by conventional clinical parameters. Expression of miR-21 tended to be reduced in lower compared to higher tumour stages and grades. Conversely, miR-126 tended to be higher in lower compared to higher tumour stages and grades. Both trends missed the level of statistical significance (Figure 2A and B).

Of 103 RCC patients in the present series, 16 showed synchronous metastasis (Table 1). In cases with synchronous metastasis versus those without, significant upregulation of miR-21 (p=0,02) and a trend towards downregulation of miR-126 missing the level of statistical significance was found (p = 0.08) (Figure 2C). But three patients with synchronous metastasis showed low miR-21 expression and notably two of these three cases had CSS exceeding three years. We concluded from these results that dysregulation of both miRs might be involved in metastasis and progression of RCC.

### Association of miR-21 and miR-126 expression with overall survival in RCC

**Figure 3:**
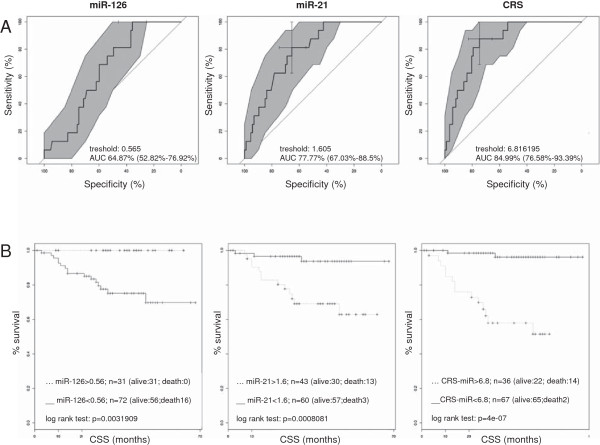
**ROC curve of miRNA risk scores (miR-21, miR-126 and CRS) and Kaplan Meier survival analysis of cancer specific survival (CSS) in RCC patients stratified by miR-21, miR-126 and CRS expression data. A)** ROC curves; the cross indicates the selected cutoff score for miR-21, miR126 or CRS resulting in highest sensitivity and specificity. The used cut-off scores were indicated in the graphs. **B)** Kaplan Meier curves with log rank test and numbers of patients stratified by the calculated risk scores of miR-21, miR-126 and CRS.

**Figure 4:**
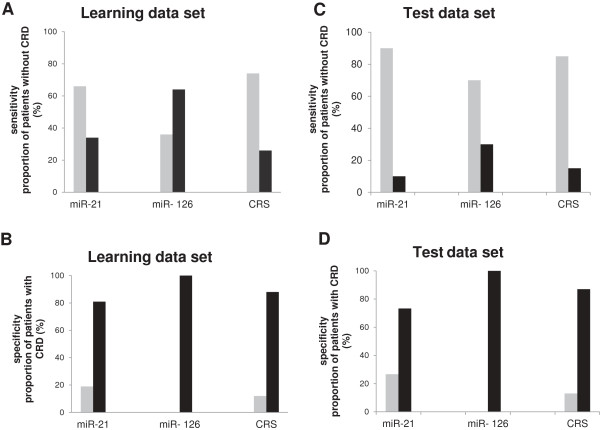
**Risk stratification of patients with or without cancer related death (CRD)*.*** Proportion of patients without **(A and C)** or with **(B and D)** cancer related death stratified by the risk score of miR-21 (high risk: miR-21 expression > 1.61), miR-126 (high risk miR-126 expression <0.57) or CRS (combined high risk score > 6.82). The proportion of correctly or incorrectly classified patients of the learning cohort **(A and B)** and the test cohort **(C and D)** are shown. The proportion of correctly classified patients at low risk **(A and B)** is shown as grey bars and indicates the sensitivity of the different risk scores. **B** and **D** show the proportion of correctly classified patients at high risk as black bars and indicate the specificity of the different risk scores. For both cohorts the CRS shows higher true positive rate and lower false positive rate as the separated miR-21 and miR-126 risk scores.

We found upregulation of miR-21 and downregulation of miR-126 to be associated with CSS, respectively (Figure 2D). The study group was dichotomized by a ROC curve and sensitivity and specificity were calculated (Figure 3A). A threshold ΔΔC_t_ = 1.61 for miR-21 and ΔΔC_t_ = 0.57 for miR-126 provided a sensitivity and specificity of 66% and 81% and of 36% and 100%, respectively (Figure 4A and B). In Kaplan-Meier analysis upregulation of miR-21 and downregulation of miR-126 were related to adverse outcome (log rank p < 0.001 for miR-21 and p < 0.01 for miR-126, Figure 3B). Expression of both miRs was combined to assess potential improvement of prediction, sensitivity and specificity. Using a previously described PCR-based risk score model CRS for CSS was calculated [19]. A cut-off of the CRS of 6.82 stratified 36 (35%) cases in the high and 67 (65%) in the low risk group, respectively.

Out of 16 cases with cancer-related death (CRD) during follow-up 14 were grouped in the predicted high risk and 65 out of 87 cases without CRD throughout follow-up in the low risk group, respectively. As shown in Figure 4 and Table 2 the CRS correctively classified 75% low risk cases and 88% high risk cases. Compared to singular miRNAs, sensitivity (88%) and specificity (75%) were increased by CRS (Figure 4A and B). Kaplan-Meier estimates showed that CRS correlated significantly with CSS (log rank p < 0.0001); predicted 5 year CSS rates were 96% for low and 48% for high risk patients, respectively.

**Table 2:** Specificity and sensitivity for the CRS in the learning (A) and test data set (B)

**A**			
**Learning data set**	**CRS > 6.8 (n)**	**CRS ≤ 6.8 (n)**	**% correct classified**
CRD (n = 16)	14	2	87,50
no CRD (n = 87)	22	65	74,71
overall			76,70
**B**			
**Test data set**	**CRS > 6.8 (n)**	**CRS ≤ 6.8 (n)**	**% correct classified**
CRD (n = 15)	13	2	86,67
no CRD (n = 20)	3	17	85,00
overall			85,71

CRD: cancer related death; CRS: combined risk score for miR-21 and miR-126.

### Prognostic model combining miR-21 and miR-126 expression

The potential of the two dichotomised miRNAs and the combined risk score (CRS) to predict overall survival in comparison to clinicopathological factors like tumour stage or grade was evaluated by uni- and multivariate Cox regression analysis. In Univariate Cox regression analysis CRS (Table 3) and both single miRs (Additional file 2: Table S2) were significantly prognostic for CSS (p < 0.0001 for CRS, p < 0.0002 for miR-126 and p < 0.0008 for miR-21; the estimate of a coefficient for miR-126 was infinity since there were no events in one group) as well as tumour stage and grade, and in contrast to age and gender, which were not significant. By stepwise regression analysis the best model for predicting CSS (tested by AIC) contained the CRS (HR: 19,37; p < 0,0002) and the clinicopathological factor tumour grade (HR: 13.88; p < 0.001) indicating that both of these factors were independent predictors of CSS in the study cohort. To internally validate this result we performed bootstrap analysis of our regression model. The bootstrap estimates generated comparable hazard ratios and confidence intervals for both factors, suggesting a robust regression model and excellent internal validation (Table 3). To evaluate the ability of miR-21 and miR-126 to predict survival we calculated a regression model by substituting the CRS with the separate dichotomised miR-21 and miR-126 expression data (Additional file 2: Table S2). Again, tumor grade and both miRs were shown to be independent factors in the regression model. Comparison of both regression models indicated that the combined risk score was a better predictor than the individual miRs.

**Table 3:** Univariate and multivariate Cox regression analysis determined by relative goodness of fit with AIC (p < 0.00001; Wald-Test) including the combined risk score (CRS) as variable

**CSS**	** Univariate**		**Multivariate (AIC)**			**Bootstrap**
	**n**	**HR (95% CI)**	**p value**	**HR (95% CI)**	**p value**	**HR (95% CI)**
CRS	103	1.107 (1.06-1.12)	2.608e-05	19.37 (4.06-92.44)	0,0002	3.97 (3.0-11.83)
Grading	103	10.97 (3.90-30.9)	5.847e-06	13.88 (4.28-45.08)	1,20e-05	3.36 (2.5-11.42)
Age	103	0.99 (0.95-1.03)	0.71			
pT	103	1.76 (1.25-2.47)	0.001			
Gender	103	1.61 (0.55-4.67)	0.38			
Wald test					p = 7.17e-07	

### Validation of the combined risk score (CRS) as prognostic factor in RCC

To validate the predictive potential of the model the determined high risk cut-off level of CRS (CRS > 6.82) was used to classify a test cohort for prognostic risk stratification. The test dataset contained 16 high risk patients with early disease progression and cancer-specific death (<48 months) and 20 low risk patients characterized by progression-free survival for over 48 months and no cancer-specific death in the follow-up time. We determined miR-21 and miR-126 expression and subsequently the CRS in samples of the validation cohort and calculated the predictive power of the CRS. Among the high risk group 13 of 15 (86.7%) and among the low risk group 17 of 20 (85%) cases were classified correctly by CRS (Figure 4C and D, Table 2). Samples of the validation cohort with CRS over the previously determined cut-off level were found to be associated with CRD by univariant Cox regression analysis (HR (95% CI) =1.39 (1.18-1.62); p < 0,0001).

## Discussion

Clinical management of RCC has changed in recent years with increased incidental diagnosis and by initiating therapy in localized stages and the establishment of antiangiogenic agents. While tumour size at time of diagnosis has decreased, mortality rate for RCC has not, suggesting an impact of differential tumour biology in morphologically similar tumours [20]. Therefore, identification of patients at high risk for cancer progression is warranted to tailor adjuvant treatment.

While numerous articles have recently studied associations between miRs and carcinogenesis and tumour progression proposing several so-called oncomiRs as regulators in carcinogenetic pathways and biomarkers in many cancer entities, considerably fewer data are available on such roles of specific miRs in RCC. Whereas recent expression studies in metastatic or progressive RCC revealed a large number of different miRs potentially linked to progression [16,21-24], surprisingly only a small number of miRs have been found to be concordantly differentially expressed in metastasised or progressive RCC. The small overlap between the different studies and largely contradictory results remains to be explained. Therefore, we selected the oncomiRs, miR-21 and miR-126, based on literature search and own unpublished expression analyses as promising markers for CSS in RCC. As expected, we identified differential expression of miR-21 in our RCC study cohort, but, surprisingly, we could not find changes in the overall miR-126 expression in our study cohort. However, miR-126 was highly up or down-regulated in subgroups of the study collective, balancing the expression changes of miR-126 in the total RCC collective. Nevertheless, the dysregulation of miR-126 in subgroups of the RCC collective might indicate a role of miR-126 dysregulation in the development of RCC. This suggestion is supported by previous studies describing an impact of miR-126 in molecular classification of different RCC subtypes [12,16,17].

Among oncogenetic miRNAs, miR-21 may be one of the most attractive for clinical use. MiR-21 is upregulated in various human cancers [4,6,25]. In vitro data support such notion; cell lines with miR-21 overexpression increase cell proliferation, migration and invasion [26]. Zhang et al. showed that knockdown of miR-21 inhibited cell proliferation and induced cell apoptosis by targeting multiple genes in RCC cells [27]. Association of miR-21 expression with adverse outcome was reported for various cancer entities, such as breast and gastric cancer [4]. Such results recently also have been reported by two previous studies using smaller RCC study cohorts [7,9]. Our present results stemming from a considerably larger and unselected series representative of tertiary cancer care are in line with these data demonstrating marked upregulation of miR-21 in RCC and significant association with synchronous metastasis and CSS. Our results show miR-21 to be an independent predictor. In one of the two previous and smaller studies in RCC Faragalla et al. found miR-21 upregulation to be associated with CSS, although it was not independent of tumour stage and grade. While Faragalla used relative miR-21 expression levels, in the present study levels of the RCC samples were normalized to adjacent benign tissue. Thus, the present study is the first to date that identifies miR-21 as an independent marker for CSS in a large and representative series using such normalization mode.

Recently miR-126 has been reported to be a tumour suppressor in various cancer types including RCC [16,17] regulating target genes like CRK, VEGF and EGFL7 in cancer cells [11]. Lately, regulation of pro-angiogenic genes has been demonstrated in metastatic breast cancer [28]. Inhibition of apoptosis in leukaemia and promotion of cancer development in NSCLC or prostate cancer have also been reported [11,29]. In several miR expression studies downregulation of miR-126 was associated with metastatic disease and early relapse after nephrectomy in smaller series of RCC [16,17]. The present series is the largest assessing miR-126 as prognostic factor in RCC to date and the first analysing expression levels normalized with benign tissue. Finding no overall downregulation of miR-126 expression we conclude no pivotal role in the initiation of RCC. Downregulation was significantly related to synchronous metastasis and independently predicted CSS. The predominant downregulation of miR-126 in progressive RCC suggest miR-126 to act as a tumour suppressor, which is supported by the recent description of miR-126 regulating VEGF-A in RCC [16], one of the pivotal factors of angiogenesis and tumour progression.

Currently, no clinically applicable molecular marker of CSS is available in RCC. To develop an accurate prediction system, we generated a dual-factorial marker model based on a CRS using the expression levels of miR-21 and miR-126. The determined CRS provided higher sensitivity and specificity compared to risk stratification, which was based on expression of each single miR. The CRS is associated with disease prognosis and predicts CSS independently from other clinicopathological factors in the analysed RCC cohort. Validation in an independent study cohort has shown that the CRS is able to classify robustly RCC samples into relevant risk groups with high sensitivity and specificity suggesting that it might have potential as a prognostic molecular assay in a clinical setting. However, the current validation is limited by various factors, like size of the validation cohort or applicability of qRT-PCR based expression analysis in clinical routine. To further test the effectiveness of this molecular marker model, we are planning to evaluate it on expanded validation cohorts in the future. Recently, a miR signature based on the expression of miR-10b, miR-139, miR-130b and miR-199b was found to be associated with synchronous metastasis and CSS in RCC [30] providing similar sensitivity (76%) and specificity (100%) as our study. Although no direct comparison can be made and the present series is considerably larger and assessed two miRs, the 5-year CSS rates predicted by both miR signatures of 32% and 84% for the high and low risk cases in the 4-miR signature compared with 48% and 96% in the current study support the robustness of such models and the impact of certain miRs on RCC. Several studies have evaluated the prognostic value of clinico-pathological features like performance status, metastatic status, lymph node involvement, sarcomatoid features, perinephritic fat invasion, Fuhrman grade and histological subtype in RCC patients [31]. We have found that only tumour grade independently predicts survival in our study cohort. Tumour stage, sex or age of the patients did not significantly correlate with prognosis and survival in our regression model. This might depend on the limited sample size and follow-up time of our RCC collective and has to be further validated in larger cohorts.

Also certain additional limitations of the present analysis need to be taken into account. For one, only clear-cell RCC was assessed limiting our conclusions to this entity. Since it represents the largest and clinically most relevant subtype, however, the clinical significance of our data is not diminished and inclusion of clear-cell RCC only added to the homogeneity of the data. Secondly, while the present series is among the largest reported to date and mode of diagnosis, surgical treatment and pathological processing are homogeneous, the data acquisition was retrospective and the exact use and regimens of anti-angiogenic medication and its impact on CSS could not be assessed. The overall use of antiangiogenic medication was homogeneously distributed over the study group and among cases with differential miR expression limiting respective bias. A further limitation is the lack of functional data; such was not the focus of the present study however, since we aimed at establishing clinical evaluation of miR as prognostic tools rather than adding basic knowledge on the role of miR in RCC tumorbiology.

## Conclusion

We found a significant correlation of miR-21-upregulation and miR-126-downregulation with metastasis and CSS in clear cell RCC. While tumour grade was the only clinicopathological parameter independently predicting CSS in the used study cohort in multivariate analysis, miR-21, miR-126 and a signature combining expression of both miRs (CRS), were independent prognosticators and might add to the limited assessment of prognosis based on clinicopathological parameters only. The determined CRS was validated in an independent test cohort showing high sensitivity and specificity in predicting CRD. The presently described miR signature appears apt to predict CSS in RCC justifying validation in larger cohorts and subsequent implication in clinical management.

## Abbreviations

miR: microRNA; RCC: Renal cell cancer; CSS: Cancer-specific survival; CRS: Combined risk score; AIC: Akaike information criterion; qRT-PCR: quantitative Real Time PCR; SD: Standard deviation.

## Competing interests

The authors declare that they have no competing interests.

## Authors’ contributions

All authors contributed to the conception and design of the study. DV conceived of this study and contributed data acquisition and interpretation, as well as drafting the manuscript. CS performed the data evaluation. SK performed the statistical analysis and helped to draft the manuscript. AR, MS, MB and HR contributed critical revision of the manuscript for scientific and factual content. BK supervised the study and guided in analysing and interpretation of the data as well as drafting the manuscript. All authors read and approved the final manuscript.

## Pre-publication history

The pre-publication history for this paper can be accessed here:

http://www.biomedcentral.com/1471-2407/14/25/prepub

## Supplementary Material

Additional file 1: Table S1 Ct levels of mir-21, miR126 und RNU6b. Click here for file

Additional file 2: Table S2 Univariate and multivariate Cox regression analysis determined by relative goodness of fit with AIC (p < 0.00001; Wald-Test) including miR-126 and miR-21 as variables. Click here for file

## References

[B1] Ljungberg B Cowan NC Hanbury DC Hora M Kuczyk MA Merseburger AS Patard JJ Mulders PF Sinescu IC EAU guidelines on renal cell carcinoma: the 2010 update Eur Urol 2010 58 3 398 406 10.1016/j.eururo.2010.06.032 20633979

[B2] Parkin DM Bray F Ferlay J Pisani P Global cancer statistics, 2002 CA Cancer J Clin 2005 55 2 74 108 10.3322/canjclin.55.2.74 15761078

[B3] Jemal A Siegel R Ward E Murray T Xu J Smigal C Thun MJ Cancer statistics, 2006 CA Cancer J Clin 2006 56 2 106 130 10.3322/canjclin.56.2.106 16514137

[B4] Hui A How C Ito E Liu FF Micro-RNAs as diagnostic or prognostic markers in human epithelial malignancies BMC Cancer 11 500 22128797 10.1186/1471-2407-11-500 PMC3260334

[B5] Catto JW Alcaraz A Bjartell AS De Vere White R Evans CP Fussel S Hamdy FC Kallioniemi O Mengual L Schlomm T MicroRNA in prostate, bladder, and kidney cancer: a systematic review Eur Urol 2011 59 5 671 681 10.1016/j.eururo.2011.01.044 21296484

[B6] Ribas J Ni X Haffner M Wentzel EA Salmasi AH Chowdhury WH Kudrolli TA Yegnasubramanian S Luo J Rodriguez R miR-21: an androgen receptor-regulated microRNA that promotes hormone-dependent and hormone-independent prostate cancer growth Cancer Res 2009 69 18 7165 7169 10.1158/0008-5472.CAN-09-1448 19738047 PMC2861586

[B7] Faragalla H Youssef YM Scorilas A Khalil B White NM Mejia-Guerrero S Khella H Jewett MA Evans A Lichner Z The clinical utility of miR-21 as a diagnostic and prognostic marker for renal cell carcinoma J Mol Diagn 2012 14 4 385 392 10.1016/j.jmoldx.2012.02.003 22580180

[B8] Juan D Alexe G Antes T Liu H Madabhushi A Delisi C Ganesan S Bhanot G Liou LS Identification of a microRNA panel for clear-cell kidney cancer Urology 2010 75 4 835 841 10.1016/j.urology.2009.10.033 20035975

[B9] Zaman MS Shahryari V Deng G Thamminana S Saini S Majid S Chang I Hirata H Ueno K Yamamura S Up-regulation of microRNA-21 correlates with lower kidney cancer survival PLoS One 2012 7 2 e31060 10.1371/journal.pone.0031060 22347428 PMC3275568

[B10] Fish JE Santoro MM Morton SU Yu S Yeh RF Wythe JD Ivey KN Bruneau BG Stainier DY Srivastava D miR-126 regulates angiogenic signaling and vascular integrity Dev Cell 2008 15 2 272 284 10.1016/j.devcel.2008.07.008 18694566 PMC2604134

[B11] Meister J Schmidt MH miR-126 and miR-126*: new players in cancer Sci World J 2010 10 2090 2100 10.1100/tsw.2010.198 PMC5763667 20953557

[B12] Youssef YM White NM Grigull J Krizova A Samy C Mejia-Guerrero S Evans A Yousef GM Accurate molecular classification of kidney cancer subtypes using microRNA signature Eur Urol 2011 59 5 721 730 10.1016/j.eururo.2011.01.004 21272993

[B13] Sasahira T Kurihara M Bhawal UK Ueda N Shimomoto T Yamamoto K Kirita T Kuniyasu H Downregulation of miR-126 induces angiogenesis and lymphangiogenesis by activation of VEGF-A in oral cancer Br J Cancer 2012 107 4 700 706 10.1038/bjc.2012.330 22836510 PMC3419968

[B14] Yang J Lan H Huang X Liu B Tong Y MicroRNA-126 inhibits tumor cell growth and its expression level correlates with poor survival in non-small cell lung cancer patients PLoS One 2012 7 8 e42978 10.1371/journal.pone.0042978 22900072 PMC3416793

[B15] Hansen TF Sorensen FB Lindebjerg J Jakobsen A The predictive value of microRNA-126 in relation to first line treatment with capecitabine and oxaliplatin in patients with metastatic colorectal cancer BMC Cancer 2012 12 83 10.1186/1471-2407-12-83 22397399 PMC3311029

[B16] Khella HW White NM Faragalla H Gabril M Boazak M Dorian D Khalil B Antonios H Bao TT Pasic MD Exploring the role of miRNAs in renal cell carcinoma progression and metastasis through bioinformatic and experimental analyses Tumour Biol 2012 33 1 131 140 10.1007/s13277-011-0255-5 22086373

[B17] Slaby O Redova M Poprach A Nekvindova J Iliev R Radova L Lakomy R Svoboda M Vyzula R Identification of MicroRNAs associated with early relapse after nephrectomy in renal cell carcinoma patients Genes Chromosomes Cancer 2012 51 7 707 716 10.1002/gcc.21957 22492545

[B18] Spahn M Kneitz S Scholz CJ Stenger N Rudiger T Strobel P Riedmiller H Kneitz B Expression of microRNA-221 is progressively reduced in aggressive prostate cancer and metastasis and predicts clinical recurrence Int J Cancer 2010 127 2 394 403 19585579 10.1002/ijc.24715

[B19] Lossos IS Czerwinski DK Alizadeh AA Wechser MA Tibshirani R Botstein D Levy R Prediction of survival in diffuse large-B-cell lymphoma based on the expression of six genes N Engl J Med 2004 350 18 1828 1837 10.1056/NEJMoa032520 15115829

[B20] Ribal MJ Molecular profiling of renal cancer: the journey to clinical application Eur Urol 2011 59 5 731 733 10.1016/j.eururo.2011.01.039 21296486

[B21] Heinzelmann J Henning B Sanjmyatav J Posorski N Steiner T Wunderlich H Gajda MR Junker K Specific miRNA signatures are associated with metastasis and poor prognosis in clear cell renal cell carcinoma World J Urol 2011 29 3 367 373 10.1007/s00345-010-0633-4 21229250

[B22] Slaby O Jancovicova J Lakomy R Svoboda M Poprach A Fabian P Kren L Michalek J Vyzula R Expression of miRNA-106b in conventional renal cell carcinoma is a potential marker for prediction of early metastasis after nephrectomy J Exp Clin Cancer Res 2010 29 90 10.1186/1756-9966-29-90 20609231 PMC2907341

[B23] White NM Khella HW Grigull J Adzovic S Youssef YM Honey RJ Stewart R Pace KT Bjarnason GA Jewett MA miRNA profiling in metastatic renal cell carcinoma reveals a tumour-suppressor effect for miR-215 Br J Cancer 2011 105 11 1741 1749 10.1038/bjc.2011.401 22033272 PMC3242591

[B24] Wotschofsky Z Liep J Meyer HA Jung M Wagner I Disch AC Schaser KD Melcher I Kilic E Busch J Identification of metastamirs as metastasis-associated microRNAs in clear cell renal cell carcinomas Int J Biol Sci 2012 8 10 1363 1374 23139634 10.7150/ijbs.5106 PMC3492794

[B25] Zhang HL Yang LF Zhu Y Yao XD Zhang SL Dai B Zhu YP Shen YJ Shi GH Ye DW Serum miRNA-21: elevated levels in patients with metastatic hormone-refractory prostate cancer and potential predictive factor for the efficacy of docetaxel-based chemotherapy Prostate 2011 71 3 326 331 10.1002/pros.21246 20842666

[B26] Lu Z Liu M Stribinskis V Klinge CM Ramos KS Colburn NH Li Y MicroRNA-21 promotes cell transformation by targeting the programmed cell death 4 gene Oncogene 2008 27 31 4373 4379 10.1038/onc.2008.72 18372920 PMC11968769

[B27] Zhang A Liu Y Shen Y Xu Y Li X miR-21 modulates cell apoptosis by targeting multiple genes in renal cell carcinoma Urology 2011 78 2 474 e413-479 10.1016/j.urology.2011.03.030 21820586

[B28] Png KJ Halberg N Yoshida M Tavazoie SF A microRNA regulon that mediates endothelial recruitment and metastasis by cancer cells Nature 2011 481 7380 190 194 10.1038/nature10661 22170610

[B29] Donnem T Lonvik K Eklo K Berg T Sorbye SW Al-Shibli K Al-Saad S Andersen S Stenvold H Bremnes RM Independent and tissue-specific prognostic impact of miR-126 in nonsmall cell lung cancer: coexpression with vascular endothelial growth factor-A predicts poor survival Cancer 2011 117 14 3193 3200 10.1002/cncr.25907 21264844

[B30] Wu X Weng L Li X Guo C Pal SK Jin JM Li Y Nelson RA Mu B Onami SH Identification of a 4-microRNA signature for clear cell renal cell carcinoma metastasis and prognosis PLoS One 2012 7 5 e35661 10.1371/journal.pone.0035661 22623952 PMC3356334

[B31] Sun M Shariat SF Cheng C Ficarra V Murai M Oudard S Pantuck AJ Zigeuner R Karakiewicz PI Prognostic factors and predictive models in renal cell carcinoma: a contemporary review Eur Urol 2010 60 4 644 661 21741163 10.1016/j.eururo.2011.06.041

