# Supplementary material for: Combination of expression levels of miR-21 and miR-126 is associated with cancer-specific survival in clear-cell renal cell carcinoma
Source: BMC Cancer. 2014 Jan 15;14:25. doi: 10.1186/1471-2407-14-25 (PMC3897948; doi:10.1186/1471-2407-14-25)
Supplement: Additional file 2: Table S2 — Univariate and multivariate Cox regression analysis determined by relative goodness of fit with AIC (p < 0.00001; Wald-Test) including miR-126 and miR-21 as variables. [file 1471-2407-14-25-S2.ppt]

## Slide 1
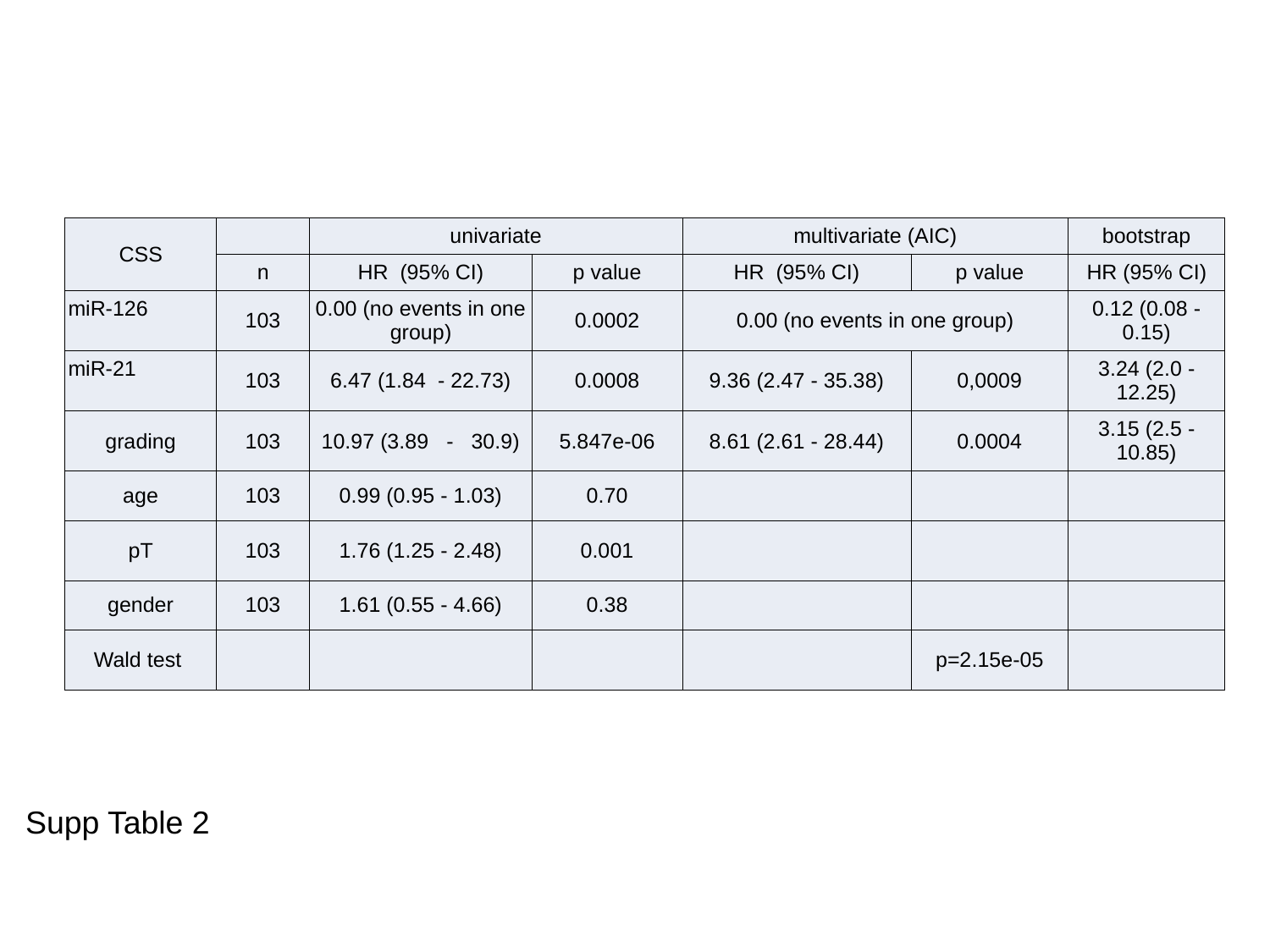

| CSS | | univariate | | multivariate (AIC) | | bootstrap |
| --- | --- | --- | --- | --- | --- | --- |
| | n | HR (95% CI) | p value | HR (95% CI) | p value | HR (95% CI) |
| miR-126 | 103 | 0.00 (no events in one group) | 0.0002 | 0.00 (no events in one group) | | 0.12 (0.08 -0.15) |
| miR-21 | 103 | 6.47 (1.84 - 22.73) | 0.0008 | 9.36 (2.47 - 35.38) | 0,0009 | 3.24 (2.0 -12.25) |
| grading | 103 | 10.97 (3.89 - 30.9) | 5.847e-06 | 8.61 (2.61 - 28.44) | 0.0004 | 3.15 (2.5 -10.85) |
| age | 103 | 0.99 (0.95 - 1.03) | 0.70 | | | |
| pT | 103 | 1.76 (1.25 - 2.48) | 0.001 | | | |
| gender | 103 | 1.61 (0.55 - 4.66) | 0.38 | | | |
| Wald test | | | | | p=2.15e-05 | |
Supp Table 2
